# Study on screening and evaluation methods of cosmetics for people with facial sensitive skin

**DOI:** 10.1097/MD.0000000000029975

**Published:** 2022-08-05

**Authors:** Jing Lyu, Qing-chun Diao, Sha Wang, Yin Yu, Yang Jiang

**Affiliations:** Department of Dermatology, Chongqing Hospital of Traditional Chinese Medicine, Chongqing, P.R. China.

**Keywords:** cosmetics, efficacy assessment, patch test, sensitive skin

## Abstract

**Objective:**

The purpose of this study is to screen and evaluate cosmetic products for sensitive skin on the face.

**Methods:**

Thirty-five subjects with positive lactic acid sting test (LAST) were recruited from the staff of our hospital from November 2019 to February 2020. First, the human skin enclosed patch test of cosmetic gel (abbreviated as gel) was performed, and then the tested products were continuously applied for 4 weeks to complete the long-term efficacy test. Subjects’ sensation of application, pruritus, tingling and burning were assessed on a 0 to 9 scale prior to, 14, and 28 days after topical application. Moreover, the transepidermal water loss rate (TEWL), stratum corneum (SC) hydration, melanin index (MI), erythema index (EI) and dendritic cells and inflammatory cell infiltration were noninvasively detected by the tester. LAST were performed before applying, 14 and 28 days after application, and then the test results were compared.

**Results:**

In this study, a total of 34 people participated in the test. The results of human skin enclosed patch test indicated that only 1 case of grade 1 reaction occurred among the tested subjects. The subjects felt good after applying the products, and the gel showed high degree of skin comfortable, no irritation and good tolerability. Subjective safety evaluation illustrated that the scores of pruritus, tingling and burning of the subjects decreased in D14 and D28 patient revisit, showing statistically significant differences (*P* < .05). When the gel was applied for 4 weeks, TEWL (8.42 ± 1.12) and EI (201.35 ± 13.51) were lower than the results before application (*P* < .05), and the SC hydration (65.36 ± 2.56) was higher than that before application (*P* < .05). There was no correlation between the SC hydration and TEWL (*R* = 0.092, *P* = .416). The results of skin CT indicated that the number of facial dendritic cells decreased in 17 subjects (accounting for 50%) in D28 patient revisit, and the degree of inflammatory cell infiltration decreased in 27 subjects (accounting for 80%). Compared with the baseline value, the LAST score and total sensory score decreased after application the product for 4 weeks, and the difference was statistically significant (the mean value of *P* < .05).

**Conclusion:**

The subjective feeling of application and efficacy of cosmetics in people with sensitive skin could be successfully evaluated by the comprehensive application of human skin enclosed patch test, long-term trial test, subjective safety evaluation and objective efficacy evaluation. And it provides the basis to judge whether the cosmetic is consistent with the efficacy claim of sensitive skin.

## 1. Introduction

Sensitive skin (SS) is the symptom of a series of skin diseases rather than an independent illness. The people suffering from SS always have conscious symptoms such as the feeling of tightness, irritation, burning, pain and pruritus,^[1,2]^ and exhibited clinical signs, such as erythema, edema, capillary dilatation, dryness, and scaling.^[3]^ Many people with SS are always stuck in the condition of cosmetic intolerance, because of which, cosmetics with soothing and repairing functions need to be used to alleviate allergic reactions and repair skin barriers. Clinically, the evaluation of the above-mentioned symptoms or signs, and the determination of objective laboratory parameters could be used to assess the applicability of a certain cosmetics for SS. In the recent years, cosmetics developed for SS are mainly aimed at repairing the barrier function of skin, alleviating skin sensitivity and inflammatory response,^[4,5]^ so as to enhance skin tolerance. In the presented paper, the variation of the following items in different periods, namely, self-feeling report, the transepidermal water loss rate (TEWL), stratum corneum (SC) hydration, melanin index (MI), erythema index (EI), dendritic cells, infiltration of inflammatory cells and lactic acid tingling test (LAST) were investigated and compared. The results are reported herein.

## 2. Materials and Methods

### 2.1. Study subjects

Thirty-five healthy women, aged from 23 to 55 (40.23 ± 11.50), were selected as the subjects. They are all employees of our hospital and indoor workers. All the selected subjects were positive for LAST at the first screening, with slight redness of face and no other skin diseases or systemic illness. No other effective skin care products, drugs, and medical beauty treatment have been applied among the subjects within 2 weeks before the test and during the test. Besides, the subjects do not take any drugs that may affect the skin state, such as antihistamines, anti-inflammatory drugs, and corticosteroid drugs. This study was approved by the Medical Ethics Committee of Chongqing hospital of traditional Chinese medicine (lot No.: 2019-ky-30), and all subjects signed the informed consents before the test.

### 2.2. Test product treatments

A.M.: cleansing→daily skin care, the tested product are used to replace the original cream.
P.M.: cleansing→daily skin care, the tested product are used to replace the original cream.
Applying area: the whole face, the dosage is the volume of 1 yuan coin each time.
Applying method: clean the face in the morning and evening. If a subject uses other skin care products normally, do not stop using them, just applying the tested products at the same time.
Use frequency and cycle: Twice a day, once in the morning and once in the evening for 28 consecutive days.

### 2.3. Product safety evaluation

The following 2 standards are referenced: GB17149.1-1997 *The General of Diagnostic Criteria and Treatment Principles of Cosmetic Dermatosis*, GB17149.2-1997 *Diagnostic Criteria and Treatment Principles of Contact Dermatitis Induced by Cosmetics*. In the experiment, the employed products were provided by a cosmetics company in China. Taking 0.020 to 0.025 g of the gel and putting it in a closed spot test device (Beijing Bai Yi Da Technology Development Co., Ltd.), and setting up the blank control (no substance was placed) at the same time. Then, the spot testers were applied to the subjects’ skin with low sensitization tape for 24 hours. In this procedure, both sides of the upper back and spine were selected as the tested locations. After that, the tested objects were removed after being applied for 24 hours. Finally, observing the skin reaction according to the standards in Table 1 and recording the observation phenomena in 3 stages, that is, 30 minutes (after the indentation disappeared), 24 hours and 48 hours after removing the tested objects spot testers.

**Table 1:** Recording standards of patch test results recommended by international contact dermatitis research group.

Judgment	Meaning	Skin manifestation
−	Negative	Normal
±	Suspicious	Mild erythema
+	Weakly positive	Erythema, infiltration, a small amount of papules
++	Strong positive	Erythema, infiltration, papule, blister
+++	Extremely strong positive	Erythema, obvious infiltration, blister and bullae

### 2.4. Subjective application feeling report and efficacy evaluation

#### 2.4.1. Subjective application feeling score.

During the return visit of D0, D14, and D28, the subjects evaluated the application feeling of the test product, including smell, stickiness, moisturization, comfort and evenness of application, and so on. The scoring standard was 0 to 9 (the greater the score, the heavier the index). Each index was expressed as the percentage of the subjects, and the total percentage of the scores more than 5 points was finally calculated.

#### 2.4.2. Clinical evaluation.

During the test duration, the professional dermatologist would grade the pruritus, tingling, and burning of the positions applied by the tested products on D0, D14, and D28, respectively. The 0 to 9 score marking system was adopted to evaluate the results (the greater the score, the heavier the level of the index). Each index was expressed as mean ± standard deviation.

#### 2.4.3. Efficacy test.

##### 2.4.3.1. The determination of skin parameters.

Mexameter MX18 (Courage & Khazaka, Germany) was used to measure the MI and EI, while Tewameter and Corneometer CM825 (Courage & Khazaka, Germany) were used to measure TEWL and SC hydration, respectively, before, 2 and 4 weeks of treatments. On the face, the measurements were taken 3 times at the same site, and the average of 3 readings was used for data analysis. All the tests were carried out under the room temperature of 22 ± 2^○^C and the relative humidity of 50 ± 5%.

##### 2.4.3.2. Determination of confocal laser scanning microscopy (also known as skin CT).

The skin CT detection was conducted when the subjects revisited on D0, D14, and D28. The state of the dendritic cells could represent the proliferative activity of inflammatory cells, hyperpigmentation, and skin sensitivity (whether it was in a highly sensitive state). The severity classification was as follows: large quantity > unequal quantity > small quantity > scattered distribution > sparse distribution. In the current status, only semiquantitative analysis could be performed for skin CT, and therefore the related images of a specific location were provided.

##### 2.4.3.3. Lactic acid tingling test.

The LAST was conducted when the subjects revisited on D0, D14, and D28, and the variations of facial skin sensitivity before and after using the product were also compared. The position of bilateral nasolabial groove was selected as the tested position. In the test, 50 µL of 10% lactic acid aqueous solution (100% lactic acid, Sigma, USA) and distilled water were pipette respectively, and then dripping them on the 2 layers filter paper for 1 minutes. Afterwards, clamping the soaked 2 layers filter paper with tweezers, placing 10% lactic acid aqueous solution on the right side of the subject’s nasolabial groove and distilled water on the left side to make it completely fit the skin. The discomfort feeling of pruritus, tingling, and burning at the tested position was evaluated by the subjects at 30 seconds, 2.5 minutes, and 8 minutes, respectively. The discomfort feeling was scored according to the 4-point marking method (0 for no feeling, 1 for mild, 2 for moderate, and 3 for severe). If the sum of tingling scores at 2.5 minutes and 8 minutes was bigger than 3, indicating that the subject was positive for lactic acid tingling.^[6]^ In case of unbearable severe tingling during the test, the test shall be stopped in time and the tested positions shall be washed repeatedly with clean water. The calculation formulas of tingling score and total sensory score were as follows^[7]^: tingling score = lactate side tingling score at 2.5 minutes and 8 minutes—distilled water side tingling score at 2.5 minutes and 8 minutes; total sensory score = all sensory scores on lactic acid side at 30 seconds, 2.5 minutes, and 8 minutes—all sensory scores on distilled water side at 30 seconds, 2.5 minutes, and 8 minutes.

### 2.5. Statistical analysis

SPSS version 21.0 (SPSS Inc., Chicago, IL) was employed for data analysis. The measurement data were expressed as mean ± standard deviation, paired t-test, and variance analysis of repeated measurement were adopted, and the nonparametric data were analyzed by Wilcoxon signed rank test. If *P* < .05, it meant that the difference was statistically significant.

## 3. Results

### 3.1. Results of enclosed patch test of human skin

One subject exhibited grade 1 reaction 24 hours after application the tested products, suggesting that the tested products had high safety among SS population, and could be applied to the later long-term trial test and efficacy test.

### 3.2. Comparison of the baseline value (D0) of each parameter and the values at different revisit time points (D14 and D28)

#### 3.2.1. Analysis of subjective application feeling.

Except for 1 subject who dropped out of the test due to his own reasons, the other 34 subjects evaluated the application feeling of the tested products when they revisited on D0, D14, and D28, the results were shown in Table 2. On return visit on D14, 58.8% of the subjects felt high degree of skin comfort and no irritation after applying the tested products; 82.4% of the subjects felt that the tested products were easy to apply and the skin was highly moisturized; 70.6% of the subjects felt that the sticky degree of the tested products was appropriate; 29.4% of the subjects felt that the smell of the tested products was irritating and smelly. In the D28 revisit, 91.2% of the subjects felt that the degree of skin comfort and moisture was high, and there was no irritation feeling; 100% of the subjects felt that the tested products were easy to apply evenly; 47% of the subjects felt that the sticky degree of the tested products was appropriate; all subjects felt that the smell of the tested products was tolerable. The above-mentioned data illustrated that with the extension of the application time, the products could be well tolerated with high security, but the smell should be further improved. During the test, 2 subjects had a little variation of acne in the D14 revisit, then we ordered them to keep a light diet and do not apply external drugs for treatment temporarily. And this symptom eventually subsided in the D28 revisit. However, it cannot be demonstrated that this symptom was directly related to the tested products due to the small sample size and limited observation time.

**Table 2:** Evaluation and analysis of feeling of application.

Proportion items		Evaluation proportion of subjects’ perception (%)										
		0	1	2	3	4	5	6	7	8	9	≥5
Irritating and unpleasant smell	D14	20.6	20.6	11.8	17.6	0.0	0.0	0.0	14.7	14.7	0.0	29.4
	D28	41.2	38.2	0.0	11.8	8.8	0.0	0.0	0.0	0.0	0.0	0.0
Greasy degree	D14	0.0	0.0	0.0	0.0	29.4	58.8	11.8	0.0	0.0	0.0	70.6
	D28	0.0	0.0	11.8	0.0	41.2	29.4	8.8	8.8	0.0	0.0	47.0
Easy to apply evenly	D14	0.0	0.0	0.0	0.0	0.0	8.8	20.6	20.6	29.4	20.6	100.0
	D28	0.0	0.0	0.0	0.0	0.0	0.0	8.8	20.6	50.0	20.6	100.0
Moisture level	D14	0.0	0.0	0.0	8.8	8.8	41.2	29.4	11.8	0.0	0.0	82.4
	D28	0.0	0.0	0.0	0.0	8.8	20.6	50	11.8	8.8	0.0	91.2
Comfort level	D14	0.0	0.0	11.8	0.0	29.4	17.6	11.8	17.6	11.8	0.0	58.8
	D28	0.0	0.0	0.0	0.0	8.8	0.0	58.8	11.8	20.6	0.0	91.2

#### 3.2.2. Analysis of clinical evaluation.

As can be seen from Table 3, compared with the baseline value, the scores of pruritus, tingling and burning decreased on D14 and D28 revisit after applying the tested products. There was no statistically significant difference in pruritus score (*P* > .05), but there were statistically significant differences in both tingling and burning (*P* < .05). It can thus be concluded that the products can alleviate the facial tingling and burning symptoms of SS, but it has no obvious efficacy in relieving pruritus.

**Table 3:** Clinical scores of subjects before and after applying the product [M (P25, P75)].

	D0	D14	D28	*P*
Pruritus	3 (2.3)	2 (1.3)	2 (2.3)	.010*
Tingling	2 (1.2)	1 (0.2)	1 (0.2)	.000*
Burning	2 (1.2)	1 (0.1)	1 (0.1)	.000*

#### 3.2.3. Results of efficacy evaluation.

##### 3.2.3.1. Results of skin parameters.

It can be seen from the Table 4 that, compared with the baseline value, the value of MI did not change obviously on D14 and D28 revisit after applying the tested products, and showed no statistically significant difference (*P* > .05); There were statistically significant differences in EI on D14 and D28 revisit (*P* < .05); When the products were applied for 4 weeks, the value of TEWL was lower than the baseline value (*P* < .05), and SC hydration was higher than the baseline value (*P* < .05), but there was no significant difference at the time point of 2 weeks (the mean value of *P* > .05). Moreover, the product could significantly reduce the EI, and its clinical manifestation was the alleviation of erythema. In contrast, the improvement effect of melanin was not obvious, and the impact on SC hydration and TEWL would be observed only after a long term of application (4 weeks). The correlation analysis between SC hydration and TEWL indicated that the value of Spearman correlation coefficient (r) was 0.092, the value of *P* was 0.416 (bilateral), demonstrating that there was no correlation between SC hydration and TEWL.

**Table 4:** Detection of facial TEWL, SC hydration, melanin, and erythema.

	TEWL (g/h/m^2^)	Stratum corneum (SC) hydration (a.u.)	Melanin index (MI)	Erythema index (EI)
D0	13.49 ± 1.19	40.98 ± 3.65	121.66 ± 6.93	286.46 ± 17.81
D14	11.05 ± 1.47	42.44 ± 3.20	120.24 ± 6.20	225.92 ± 11.65∆
D28	8.42 ± 1.12∆	65.36 ± 2.56∆	122.10 ± 5.63	201.35 ± 13.51∆
Value of F	12.69	24.32	2.89	39.86
Value of P	.029*	.018*	.459	.001*

##### 3.2.3.2. Results of skin CT.

**Figure 1.**
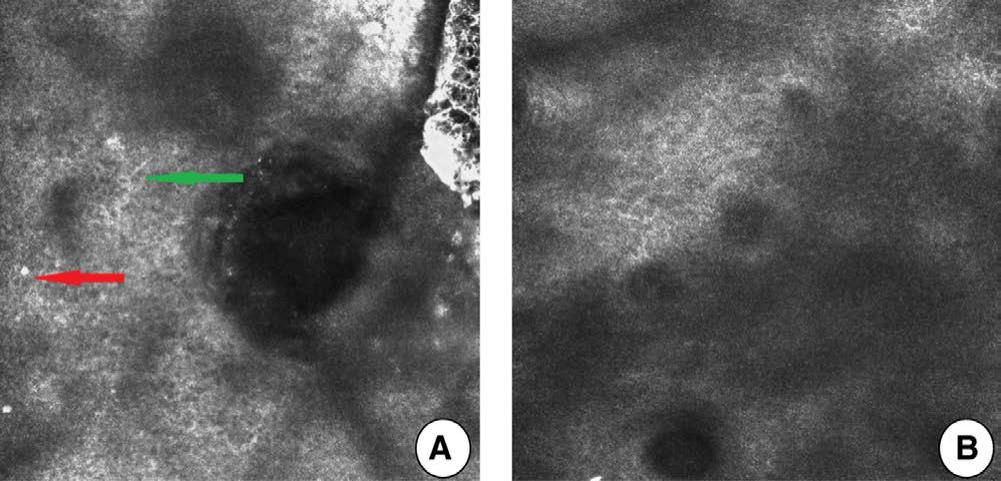
Images of skin CT after applying the tested products on D0 and D28. (A) CT analysis of facial skin of a subject before using the tested product: mild edema between prickle cells, unequal amount of dendritic cells (green arrow) can be observed in the basal layer, telangiectasia, and congestion occurred in the superficial dermis, scattered distribution, and unequal amount of inflammatory cells infiltration (red arrow) can be observed around the tube, partial hair follicles dilated and occurred keratotic plug, and no hair follicle worm was found in it. (B) No obvious dendritic cells and inflammatory cells could be found 28 days after using the tested products.

The images of skin CT analysis were shown in Figure 1A, B. The results indicated that the number of dendritic cells decreased in 17 subjects (accounting for 50%) on the D28 revisit, and the degree of inflammatory cell infiltration alleviated in 27 subjects (accounting for 80%). Moreover, 1 subject had a slight increase in dendritic cells, which was considered to be related to the increase of pigmentation after inflammation. Furthermore, inflammatory cells increased slightly in 2 subjects, but there was no obvious clinical manifestation. The decrease of the infiltration degree of dendritic cells and inflammatory cells suggested the decrease of skin sensitivity, and this symptom can be improved only after a long term of applying.

##### 3.2.3.3. Results of lactic acid tingling test.

The variance analysis of repeated measurement was employed in the LAST, and the results were listed in Table 5. The results indicated that there were differences in the LAST at different time points. It can be deduced by LSD comparison method that the LAST score and total sensory score after applying the products for 4 weeks were significantly lower than the baseline values (as can be seen from Table 5). The results suggested that the tested products had the ability to increase skin tolerance and reduce sensitivity during the test cycle, which may be attributed to the function of repairing skin barrier.

**Table 5:** Comparison of LAST score and total sensory score at different time points[M (P25, P75)].

Observation index	D0	D14	D28	*P*
Tingling score	4 (3.5)	3 (3.5)	1 (1.2)	.000*
Total sensory score	7 (5.8)	7 (5.8)	3 (3.4)	.000*

## 4. Discussion

Sensitive skin is a skin status with increased local reactivity, and it is commonly found in the faces of females. The skin of specific parts of this population is sensitive to slight conventional external stimulation, and therefore, they always can not tolerate or reduce tolerance to commonly applied cosmetics. The main physiological and pathological mechanism of SS is the reduction of the “threshold” of the external stimulation, resulting in a response to a slight external stimulation. However, this slight external stimulation will not cause skin reactions under normal skin conditions most of the time.^[8]^ Acne, rosacea, seborrheic dermatitis, corticoid-dependent dermatitis and other facial skin diseases are often complicated with various degrees of skin sensitivity, and repairing the skin barrier function is also a very important link throughout the treatment process of this kind of disease. Due to the fact that the SC barrier function is damaged and the skin repair function is poor for SS population, it is necessary for them to choose cosmetics with soothing and repairing effect. In this kind of products, not only the ingredients that may cause skin irritation or allergy should be reduced, but also effective ingredients that could promote skin barrier repair, inhibit inflammatory reaction and nerve sensitivity should be incorporated.^[9]^ Currently, there are no international standard method for the efficacy test of SS applicable cosmetics. Therefore, the methods presented in this paper were formulated based on literature and our own experimental conditions. In this method, the subjective and objective methods were combined, so as to evaluate the applicability of the cosmetic product for SS.

The SC hydration and TEWL are 2 important parameters to evaluate the barrier function of SC. When the skin barrier function was damaged, the SC hydration decreased but the TEWL increased, resulting in an increase in the transdermal osmolality of irritants or antigens, which therefore inducing irritation or immune response, and symptoms such as obvious tingling, burning, and pruritus. However, the barrier repairing agent could facilitate the skin barrier function by reducing TEWL and improving SC hydration in a short period. The noninvasive method used in this experiment to detect the physiological parameters of the skin has high sensitivity and repeatability. It mainly measures the superficial SC hydration.^[10]^ Besides, TEWL can reflect the water loss of the SC and is an important parameter for evaluating the SC barrier. The combination of the above 2 parameters would better reflect the repair function of the tested products on the skin barrier function. As an important test method to judge SS, LAST could also be used to evaluate the severity of SS. LAST, TEWL, and SC hydration were always employed to evaluate SS barrier function in clinical studies.^[7]^

The results of this study showed that there was no significant change in the score of LAST at 2 weeks after applying the test product, but the score of LAST at 4 weeks exhibited a downward trend, significantly lower than the baseline (*P* < .05), indicating that the product can not reduce the stinging feeling in a short time, and it will be significantly improved if it was applied for 4 weeks or longer time. The efficacy evaluation demonstrated that TEWL decreased significantly and SC hydration increased markedly 2 or 4 weeks after applying the products, and there was statistically significant difference before and after the treatment. After applying the products for 4 weeks, the EI decreased obviously, suggesting that the degree of vascular dilatation was alleviated, and the clinical symptoms of burning and tingling were also improved. Dendritic cells, as most powerful antigen-presenting cells in human body, may be of great significance in various inflammatory skin diseases, such as atopic dermatitis, psoriasis, and contact dermatitis. Moreover, it serves as an indicator to indirectly reflect the sensitivity of the skin.^[11]^ The results of skin CT examination indicated that the number of dendritic cells decreased in 15 subjects (accounting for 50%) after applying the product on D28, and the degree of inflammatory cell infiltration decreased in 24 subjects (accounting for 80%). The above results demonstrated that the tested products could accelerate the repair of skin barrier function, increase SC hydration, lower the TEWL and EI, as well as enhance the tolerance to external stimulation.

During the test period, the closed patch test showed that only 1 subject exhibited grade 1 reaction 24 hours after applying the test product, indicating that the product has high safety. From the subjective evaluation, 35 subjects were highly satisfied with the test products. Compared with the baseline value, the scores of itching, tingling, and burning of the subjects decreased on D14 and D28 revisits. It was considered that the product had a certain effect on improving skin sensitivity, but the itching score did not show a statistically significant difference (*P* > .05), which may be related to the fact that the itching symptoms of the subjects were not so obvious.

Studies have shown that the impaired skin barrier function is an important pathogenesis of SS.^[12,13]^ Currently, the research on SS treatment at home and abroad focuses on the application of medical skin care products to repair the damaged skin barrier. A clinical study was conducted to observe the tolerance of 94 patients with a history of contact allergy to sterile cleaners and moisturizers free of emulsifier, preservative, and fragrance. The results indicated that the irritation and all objective symptoms of SS were significantly improved, suggesting that properly formulated medical skin care products could improve SS symptoms such as dryness, erythema and tingling.^[14]^ Hawkins et al^[15]^ evaluated the benefits of daily facial skin care programs containing mild cleansers and moisturizers for SS patients. Through doctor evaluation, instrument evaluation, and subjective self-evaluation, it was observed that facial skin sensitivity decreased markedly and the skin health improved significantly. In the presented study, the adopted evaluation method is suitable for judging the applicability of the tested products for SS population with high scientificity, operability, and repeatability. The obtained results can not only provide guidance for consumers to choose cosmetics, and provide theoretical basis for cosmetics manufacturers to improve product formula, but also provide efficacy determination basis for cosmetics claiming to be “suitable for sensitive skin.”

Nevertheless, there are also limitations should be further improved. For example, the results of multi probe skin test are vulnerable to the testing environment, testing posture and other factors; the subjects may not use the product according to the requirements. Thus, it is necessary to further strengthen the supervision and control the test conditions.

## Author contributions

Yang Jiang and Qing-chun Diao conceived and planned the study, interpreted the data, and revised the manuscript. Jing Lyu analyzed the data and drafted the manuscript. Sha Wang, Yin Yu completed the test and data collection.

## Footnotes

* Positive rate was significant difference between D0 value and D28.

* Indicates that there is difference between the 3 groups.

∆Indicates that there is difference as compared to the baseline value.

* Positive rate was significant difference between baseline value and 4 weeks.
